# Protective Effects of Curcumin and Sulforaphane Against Ionising Radiation–Induced Oxidative Stress and Inflammatory Responses in Rat Lung Tissue

**DOI:** 10.3390/antiox15020255

**Published:** 2026-02-15

**Authors:** Katarina Baralić, Jovana Živanović, Predrag Božović, Nikola Kržanović, Neda Šćepanović, Jelena Petrović, Danijela Đukić-Ćosić

**Affiliations:** 1 Department of Toxicology “Akademik Danilo Soldatović”, Faculty of Pharmacy, University of Belgrade, Vojvode Stepe 450, 11221 Belgrade, Serbia; jovana.zivanovic@pharmacy.bg.ac.rs (J.Ž.); neda00scepanovic@outlook.com (N.Š.); pjelena600@gmail.com (J.P.); danijela.djukic.cosic@pharmacy.bg.ac.rs (D.Đ.-Ć.); 2 Department of Radiation and Environmental Protection, Vinča Institute of Nuclear Sciences, University of Belgrade, 11000 Belgrade, Serbia; bozovic@vinca.rs (P.B.); krzanovic@vinca.rs (N.K.)

**Keywords:** oxidative stress, inflammation, γ-irradiation, antioxidants, phytochemicals

## Abstract

Ionising radiation-induced lung injury is a major complication of thoracic radiotherapy, primarily driven by oxidative stress and inflammation. The current study evaluates and compares the protective effects of sulforaphane (SFN) and curcumin (CUR) pretreatment against radiation-induced oxidative damage and inflammation in rat lung tissue. Female *Wistar* rats were pretreated in vivo with SFN (2 mg/kg b.w./day) or CUR (4.13 mg/kg b.w./day) for 28 days per os. Isolated lung tissues were exposed ex vivo to γ-radiation (absorbed dose: 2 Gy). Oxidative stress markers—malondialdehyde (MDA), ischemia-modified albumin (IMA), total sulfhydryl (SH) groups, reduced glutathione (GSH), and superoxide dismutase (SOD)—and inflammatory markers—tumour necrosis factor alpha (TNF-α), prostaglandin-endoperoxide synthase 2 (PTGS2/COX-2), interleukin-6 (IL-6), and interleukin-1 beta (IL-1β)—were measured to evaluate irradiation and protective effects. Radiation significantly increased MDA, TNF-α, PTGS2/COX-2, and IL-6 levels while decreasing SH groups. Pretreatment with SFN or CUR attenuated these changes. CUR showed a more pronounced effect on oxidative stress-related parameters, whereas SFN more strongly influenced inflammatory markers. These findings suggest that SFN and CUR differentially modulate radiation-induced oxidative and inflammatory responses in lung tissue under the applied experimental conditions and warrant further investigation of their potential as protective agents in radiotherapy.

## 1. Introduction

Radiotherapy is a fundamental modality in oncology, applied in over 50% of cancer patients. It offers definitive, organ-preserving therapy in early-stage tumours and supports curative treatment in locally advanced disease, either alone or with systemic therapy. It can also be administered perioperatively to improve surgical outcomes [1]. However, the biophysical actions of radiotherapy are not limited to malignant cells and can cause toxicity in adjacent healthy tissues and organs [2]. Within the electromagnetic spectrum, ionizing radiation includes X-rays, with wavelengths between 0.01 and 10 nm, and gamma rays, which have wavelengths below 0.001 nm. Additionally, natural sources such as radon gas and cosmic rays contribute to a background exposure averaging about 2.4 mSv per year [3]. Ionizing radiation triggers chemical, molecular, cellular, and tissue-level events that drive radiation sickness and organ injury. It ionizes water and oxygen, generating free radicals that induce oxidative and nitrosative stress and damage nuclear DNA, while secondary electrons create additional ionizations and further DNA injury [4].

Radiation-induced lung injury (RILI) is a major complication of thoracic radiotherapy and represents a significant risk for patients undergoing total-body irradiation [5]. Over half of patients with breast or other thoracic tumours receive radiotherapy. Its expanding use in palliative settings and oligometastatic disease further increases the population at risk for RILI. RILI incidence varies by tumour type, affecting 5–25% of lung cancer patients, 5–10% of mediastinal lymphoma patients, and 1–5% of breast cancer patients [6]. Radiation triggers lung responses such as pneumonitis and fibrosis, driven by excessive free-radical generation and sustained alterations in immune mediators [5]. RILI comprises two phases. Acute radiation pneumonitis arises within weeks to six months and may be life-threatening when extensive. The late phase occurs months to years later, characterized by reduced inflammation and progressive collagen deposition, leading to irreversible pulmonary fibrosis [7]. In clinical radiotherapy, normal tissue tolerance limits both dose and treated volume. Therefore, maximum doses rarely exceed 70 Gy. Most microscopic disease can be controlled with 40–50 Gy, many gross tumours with 60–65 Gy, while higher-risk tumours may require modest dose escalation beyond these levels [8]. The amount of radiation the lungs receive is the strongest predictor of radiation pneumonitis. The risk increases when larger portions of the lung receive 20 Gy or more, or even lower doses such as 5–13 Gy [9]. In rats, irradiation at doses of 1 Gy or higher resulted in a significant, dose-dependent reduction in type II pneumocytes as well as an increase in alveolar neutrophils [10]. The mean lung dose is the most reliable predictor of severe radiation pneumonitis (grade > 3), with even small increases raising the risk of clinically significant toxicity [9].

Limiting lung exposure is, therefore, the primary strategy to reduce severe RILI. However, effective radioprotective agents could provide additional protection. Plant-derived secondary metabolites, including flavonoids, phenylpropanoids, resveratrol, quercetin, ascorbic acid, and gallic acid, exert radioprotective effects via antioxidant, anti-inflammatory, and cytoprotective mechanisms [11]. Accordingly, given the central role of oxidative stress and inflammation in RILI, natural compounds with antioxidant and anti-inflammatory properties, such as sulforaphane (SFN) and curcumin (CUR), have been explored for radioprotection. SFN is an isothiocyanate found in cruciferous vegetables such as broccoli. This compound has been widely studied for its health-promoting and anticancer properties. It exerts antitumor effects in several malignancies, including breast, lung, bladder, colon, prostate, and kidney cancers. These effects are mediated through the regulation of cell cycle progression, induction of apoptosis, and modulation of metabolic enzymes [12]. A key protective mechanism of SFN is the activation of the Nrf2 signalling pathway via the modification of Keap1 cysteine residues, leading to an increased expression of cytoprotective genes. This activation enhances cellular defence against ROS and electrophiles while downregulating inflammatory processes [13]. SFN has been reported to mitigate RILI by suppressing the inflammatory mediator NLR family pyrin domain containing 3 (NLRP3) [14]. This complex acts as an upstream regulator of NF-κB signalling and is involved in controlling inflammation, immune responses, and apoptosis [15]. CUR, a natural biphenolic compound found in turmeric, exhibits antioxidant, anti-inflammatory, and anticancer properties in various animal models [16,17]. In lungs, CUR exerts its effects by reducing oxidative stress, proinflammatory and fibrogenic cytokines, and NF-κB expression, with these processes occurring both concurrently and in sequence [18]. CUR’s radioprotective effects primarily involve the attenuation of oxidative stress, inflammatory responses, and fibrotic processes [5,19,20,21].

To the best of our knowledge, while CUR’s radioprotective effects in the lung are supported by a limited number of animal studies [5,19,20,21], SFN has been scarcely investigated, with only one experimental study to date [14]. To address this knowledge gap, we directly compared CUR and SFN pretreatment to assess their protective potential against radiation-induced oxidative stress and inflammation parameters in lung tissue. We hypothesized that pretreatment with either compound modulates redox balance and inflammatory status, thereby attenuating radiation-induced damage. Accordingly, the aim of the present study was to evaluate and directly compare the radioprotective effects of CUR and SFN in a rat lung tissue model exposed to ionizing radiation.

## 2. Materials and Methods

### 2.1. Chemicals

This study used curcumin (Sigma Aldrich, St. Louis, MO, USA), sulforaphane (≥90% D,L-Sulforaphane, Santa Cruz Biotechnology, Dallas, TX, USA), and dimethyl sulfoxide (DMSO, 99.9%, Fisher Chemical, Loughborough, UK). For the measurement of oxidative stress parameters, thiobarbituric acid (TBA), cobalt chloride (CoCl_2_), 5,5′-dithiobis(2-nitrobenzoic acid) (DTNB), sulfosalicylic acid, and adrenaline (epinephrine), were purchased from Merck (Darmstadt, Germany). Dithiothreitol (DTT), Bradford ready-to-use dye reagent, and bovine serum albumin standard were obtained from Fisher Scientific (Waltham, MA, USA).

### 2.2. Experimental Animals

Female Wistar rats (*Rattus norvegicus*), 6–8 weeks old and weighing 150–200 g, were obtained from the Experimental Animal Facility of the Military Medical Academy in Belgrade, Serbia. All animals were clinically healthy and had not undergone any previous experimental procedures prior to inclusion in this study. The genotype was wild type. The rats were housed in plastic cages with wire tops under controlled environmental conditions (temperature of 20–24 °C, relative humidity of 35–60%, and 12 h/12 h light–dark cycle). Animals had unrestricted access to drinking water and standard laboratory chow (Veterinary Institute “Subotica,” Subotica, Serbia), a commercially available rodent diet, throughout this study. Standard environmental enrichment was provided in the form of nesting material. All experimental procedures were approved by the Ethics Committee for Animal Experimentation of the Faculty of Pharmacy, University of Belgrade, and by the Ministry of Agriculture, Forestry, and Water Management—Veterinary Directorate (permit number: 001748492 2025). Experiments were conducted in accordance with the Faculty of Pharmacy’s guidelines for animal care and use. Only female rats were used in this experiment because thoracic irradiation is more commonly performed in females. This is mainly due to breast carcinoma, a highly prevalent malignancy that often requires radiotherapy in the thoracic region [22], which frequently results in incidental lung exposure. The decision also aligned with the 3R principle (Replacement, Reduction, Refinement), with the aim of reducing the number of experimental animals used. Rats were chosen as an appropriate experimental model considering that the structural and functional organization of rodent lungs closely resembles that of larger mammals, differing primarily in size, which supports their use as models of the human lung [23].

### 2.3. Experimental Protocol

No pre-registered experimental protocol was prepared for this study. A total of 15 animals were used, with 5 rats allocated to each experimental group. The number of animals was determined in accordance with OECD Test Guideline No. 407 (Repeated Dose 28-Day Oral Toxicity Study in Rodents), and the statistical power of this study was additionally verified using G*Power software 3.1 (Heinrich Heine University, Düsseldorf, Germany). Animals were randomly allocated to experimental groups using a simple randomization procedure, whereby each animal was assigned to a group based on a computer-generated random sequence. The rats were divided into three groups: a control and two treated groups (SFN and CUR). SFN dose (2 mg/kg b.w./day) was selected based on prior dose-conversion calculations conducted by our research group using the U.S. Food and Drug Administration (FDA) human-to-rat formula, corresponding to doses commonly found in dietary supplements in Serbia, which yielded an estimated equivalent of 5 mg/kg b.w./day. Although higher doses were tested in our previous study, 2 mg/kg showed a more favourable efficacy–safety profile across biochemical, haematological, redox, histopathological, and inflammatory parameters. Therefore, this dose was chosen for the present study as a biologically effective, well-tolerated regimen [24]. The CUR dose was determined using the FDA human-to-animal dose conversion formula corresponding to doses commonly found in commercially available dietary supplements in Serbia. Assuming an average human body weight of 60 kg, the equivalent rat dose was calculated as: 40 mg/day ÷ 60 kg/day × 6.2 = 4.13 mg/kg b.w./day; the coefficient 6.2 was used for extrapolation from humans to rats. SFN and CUR were dissolved in 100 mL deionized water with the addition of 5 mL DMSO (5% solution). DMSO is considered an appropriate solvent for studies of antioxidant activity when used at low concentrations (≤5%), as it does not confound the assessment of lipophilic compounds in redox status assays, and is widely accepted in redox status studies [25]. The control group received 5% DMSO solution for 28 days administered at a volume of 1 mL/kg body weight. The use of a vehicle control ensured that any observed effects could be attributed to the test compounds rather than to the solvent itself. Rats were administered CUR and SFN daily for 28 days by oral gavage. Animals were weighted daily throughout the 28-day treatment period, and dosing volumes were adjusted accordingly to ensure accurate administration of CUR and SFN based on the current body weight. Animals were monitored daily for general health status, including activity, posture, grooming behaviour, food and water intake, and signs of distress or discomfort. No adverse effects related to treatment were observed. No specific randomization of the order of measurements or blinding of investigators was applied, and animal or cage location was not considered as a controlled variable. No predefined humane endpoints were required, as no animals exhibited clinical signs necessitating early euthanasia. Predefined inclusion and exclusion criteria were applied. Animals of the specified body weight range that were clinically healthy and completed the experimental protocol were included. Exclusion criteria comprised illness unrelated to treatment, technical or procedural errors, anaesthesia-related complications, or inadequate tissue quality. No animals nor tissues met the exclusion criteria, and all were included in the final analysis. No expected or unexpected adverse events occurred during this study. Blinding was not implemented during experimental procedures, outcome assessment, or data analysis. Group allocation was known to the investigators at all stages of the experiment. Allocation to experimental groups was performed by the researchers conducting this study. The primary outcome measures of this study were oxidative stress parameters and inflammatory markers assessed in non-irradiated and irradiated lung tissue. After 28 days of exposure, the animals were deeply anesthetized by intraperitoneal injection of ketamine and xylazine (75 and 10 mg/kg, respectively) and euthanized by cardiac puncture, after which the lungs were extracted, snap-frozen in liquid nitrogen, and stored at −80 °C until further processing and analysis. The experimental unit was a single animal for the in vivo pretreatment phase, and the isolated lung tissue sample obtained from each animal for ex vivo irradiation and subsequent analyses. Left and right lungs from the same rats were used as paired biological samples to minimize inter-animal variability. To account for anatomical asymmetry between the lungs, isolated lung tissue was divided into equivalent portions, and assignment to irradiated or non-irradiated conditions was performed randomly. This paired ex vivo design enabled a direct comparison of radiation effects and radioprotective interventions within the same biological background. The experimental design included six final groups: Control: lung tissue from vehicle-treated rats (5% DMSO, 28 days); SFN: lung tissue from rats pretreated with SFN (2 mg/kg/day, 28 days); CUR: lung tissue from rats pretreated with CUR (4.13 mg/kg/day, 28 days); RAD: lung tissue from control rats ex vivo exposed to radiation (absorbed dose: 2 Gy); RAD + SFN: lung tissue from SFN-pretreated rats ex vivo exposed to radiation (absorbed dose: 2 Gy); RAD + CUR: lung tissue from CUR-pretreated rats ex vivo exposed to radiation (absorbed dose: 2 Gy). These six final experimental groups (control, RAD, SFN, CUR, RAD + SFN, and RAD + CUR) were compared to evaluate both the effects of ionizing radiation on lung tissue and the potential protective effects of sulforaphane and curcumin pretreatment.

### 2.4. Sample Handling and Lung Tissue Exposure to Ionizing Radiation

In compliance with the Serbian Animal Welfare Act (“Official Gazette RS”, No. 41/2009), which prohibits direct exposure of live animals to radiation, an ethically and legally acceptable alternative was used: isolated lung tissue samples were irradiated instead of live animals. Isolated lung tissue samples were stored at −80 °C until use and transported to the Vinča Institute of Nuclear Sciences on dry ice. Immediately prior to irradiation, samples were thawed and maintained on ice (0–4 °C) to enable radiolytic processes. Irradiation was performed in an unfrozen state at the Institute. Following irradiation, samples were immediately snap-frozen on dry ice and transported in insulated containers to the Faculty of Pharmacy, University of Belgrade. Throughout transport, samples were kept frozen to prevent degradation and artificial oxidation. Oxidative stress parameters and inflammatory markers were analysed at the Faculty of Pharmacy on the same day as irradiation, avoiding additional freeze–thaw cycles. Redox status and inflammatory parameters were assessed using five independent biological samples per experimental group, with each sample measured once. Due to limited sample material, all assays were performed as single measurements. To minimize analytical variability, samples were processed under identical conditions, and all measurements were conducted within the same analytical run. The irradiation process was carried out in the Radiation and Environment Protection Department of Vinča Institute of Nuclear Sciences, an accredited secondary standard dosimetry laboratory. Tissues were irradiated using gamma photons from cobalt-60 source (nominal activity A = 238 TBq, as of September 1999). The reference dose field was established using calibrated ionization chambers traceable to the primary dosimetry standards of the International Atomic Energy Agency (IAEA). A fixed source-to-sample distance of 70 cm was used with the cobalt unit shutter at maximum opening to ensure uniform irradiation. An exposure time of 30 min was selected to deliver a total absorbed dose of 2 Gy to the tissue samples. All tissue samples from different experimental groups were irradiated in parallel under identical experimental conditions to minimize potential confounding effects related to processing order or environmental factors. The absorbed dose of 2 Gy corresponds to a therapeutic dose that can be administered in a single clinical session without significant damage to surrounding healthy tissue [26]. While this dose is lower than those typically causing severe pneumonitis in patients, it is sufficient to induce oxidative stress and DNA damage in isolated lung tissue. This approach allows the investigation of early molecular and cellular responses to radiation and the evaluation of potential radioprotective effects of SFN and CUR in a controlled, reproducible setting.

### 2.5. Oxidative Stress—Sample Preparation and Parameter Determination

Lung tissue samples (irradiated and non-irradiated) were homogenized using a T10 basic UltraTurrax homogenizer (IKA, Königswinter, Germany) in 0.1 mol/L phosphate buffer (pH 7.4; weight-to-volume ratio 1:9). Homogenates were centrifuged at 800 *g* for 10 min, followed by 9500 *g* for 10 min to obtain postmitochondrial supernatants. Centrifugation was carried out using a centrifuge equipped with a cooling system (Eppendorf Centrifuge 5417R, Eppendorf, Hamburg, Germany). The supernatants were stored at −80 °C until analysis.

The following oxidative stress markers (malondialdehyde (MDA) and ischemia-modified albumin (IMA)) and antioxidant defence parameters (total sulfhydryl (SH) groups, glutathione (GSH) levels, and superoxide dismutase (SOD) activity) were determined. All reagents were of analytical grade and obtained from commercial suppliers. Measurements were performed using a SPECTROstar Nano UV/VIS spectrophotometer (BMG Labtech, Ortenberg, Germany). Calibration curves were constructed to calculate the concentrations of oxidative stress and antioxidant markers. Table 1 summarizes the methodology used for each parameter. Protein concentration was determined using the Bradford dye-binding assay with bovine serum albumin (BSA) as the standard, and enzyme activities were normalized to mg protein.

Three scores were calculated using measured oxidative stress/antioxidant defence parameters: damage score (DS), protection score (PS), and OXY score (overall oxidative balance). DS was calculated as the mean of pro-oxidative markers (MDA and IMA, expressed as z-scores), while PS was calculated as the mean of antioxidant markers (SH groups, GSH, and SOD). OXY score was obtained by subtracting PS from DS. An OXY score of zero indicates balance between oxidative damage and antioxidant protection, i.e., elevated oxidative damage is compensated by high antioxidant activity [32].

### 2.6. Inflammatory Biomarkers

The levels of TNF-α, PTGS2, IL-6, and IL-1β in rat lung tissue homogenates were quantified using commercially available enzyme-linked immunosorbent assay (ELISA) kits (ELK Biotechnology, Sugar Land, TX, USA), following the manufacturer’s protocols. Lung tissue homogenates were prepared and appropriately diluted to fall within the dynamic range of each assay. Samples, standards, and controls were added to pre-coated microplate wells and incubated to allow binding of the target cytokines to specific antibodies. After washing to remove unbound components, enzyme-linked secondary antibodies were added, followed by a substrate solution that produced a colorimetric signal proportional to the cytokine concentration. Absorbance was measured at the specified wavelength using a SPECTROstar Nano UV/VIS spectrophotometer (BMG Labtech, Ortenberg, Germany). Cytokine concentrations in the samples were determined by comparison to standard curves generated for each analyte.

### 2.7. Statistical Analysis

Data analysis and visualization were performed by GraphPad Prism 10 (GraphPad Software, Inc., San Diego, CA, USA). The Shapiro–Wilk test was applied to assess data normality. As all variables followed a normal distribution, one-way analysis of variance (ANOVA) was applied, followed by Tukey’s post hoc multiple-comparison test to compare all experimental groups with each other. Statistical significance was set at *p* < 0.05. For all statistical analyses, the exact sample size was *n* = 5 per experimental group.

## 3. Results

In the following text, only statistically significant differences between experimental groups are described for clarity and conciseness; all six final experimental groups (control, SFN, CUR, RAD, RAD + SFN, and RAD + CUR) were included in the analyses and are shown in the figures, but non-significant comparisons are not discussed unless no differences were observed among any of the groups.

**Figure 1:**
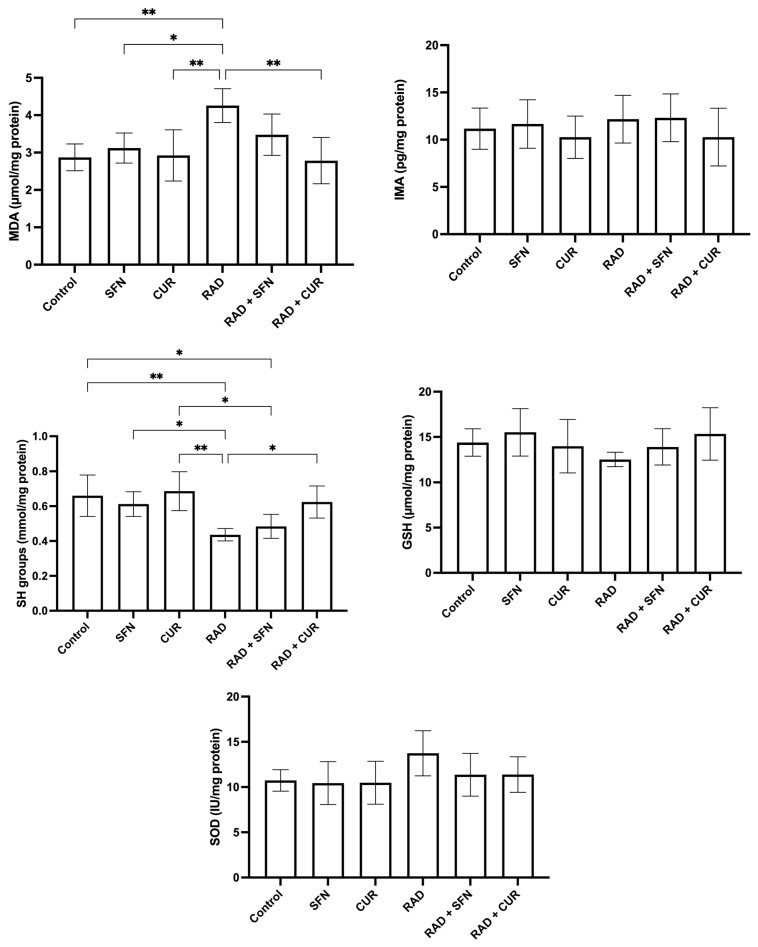
The effect of a single exposure of rat lung tissue to ionizing radiation on parameters of oxidative stress and antioxidant protection, as well as the protective effects of sulforaphane (SFN) and curcumin (CUR) administered per os for 28 days prior irradiation. Results are expressed per mg of protein (Bradford assay, bovine serum albumin standard). Control: lung tissue from vehicle-treated rats (5% DMSO, 28 days); SFN: lung tissue from rats pretreated with SFN (2 mg/kg/day, 28 days); CUR: lung tissue from rats pretreated with CUR (4.13 mg/kg/day, 28 days); RAD: lung tissue from control rats ex vivo exposed to radiation (absorbed dose: 2 Gy); RAD + SFN: lung tissue from SFN-pretreated rats ex vivo exposed to radiation (absorbed dose: 2 Gy); RAD + CUR: lung tissue from CUR-pretreated rats ex vivo exposed to radiation (absorbed dose: 2 Gy). Statistical analysis: one-way ANOVA + Tukey’s post hoc multiple-comparison test. Statistical significance is denoted by asterisks: * *p* < 0.05, and ** *p* < 0.01. Data are presented as mean ± SD. Bars represent mean values, and error bars indicate ± SD. *n* = 5 independent biological samples per group. Horizontal lines indicate the specific pairwise comparisons between the groups; comparisons without connecting lines are not statistically significant.

The measured parameters of oxidative stress and antioxidant protection in rat tissue homogenates are shown in Figure 1.

MDA levels were significantly increased in the RAD group compared with the control group (*p* < 0.01), SFN-treated animals (*p* < 0.05), and CUR-treated animals (*p* < 0.01). Among irradiated groups, RAD + CUR exhibited significantly lower MDA levels compared with RAD alone (*p* < 0.01).

SH group levels were significantly decreased in the RAD group compared with the control group (*p* < 0.01), SFN-treated animals (*p* < 0.05), and CUR-treated animals (*p* < 0.01). Within irradiated groups, RAD + CUR showed significantly higher SH levels compared with RAD alone (*p* < 0.05), while SH levels were significantly lower in RAD + SFN compared with CUR (*p* < 0.05). A significant decrease in SH levels was observed in the RAD + SFN group compared to the control (*p* < 0.05).

For SOD, no statistically significant differences were observed between the experimental groups, although a visible trend toward increased levels was noted in the RAD group.

In the case of GSH and IMA, there was no observed significant changes in none of the treated groups compared to the control.

**Figure 2:**
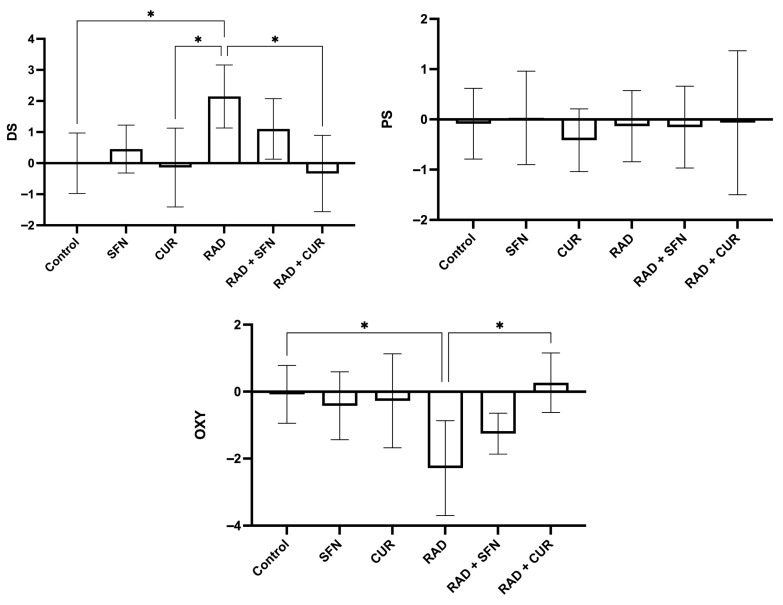
Calculated scores of oxidative stress (damage score, DS; protection score, PS; and OXY score, overall oxidative balance score) in rat lung tissues after single exposure to ionizing radiation and the protective effects of sulforaphane (SFN) and curcumin (CUR) administered per os for 28 days prior irradiation. Control: lung tissue from vehicle-treated rats (5% DMSO, 28 days); SFN: lung tissue from rats pretreated with SFN (2 mg/kg/day, 28 days); CUR: lung tissue from rats pretreated with CUR (4.13 mg/kg/day, 28 days); RAD: lung tissue from control rats ex vivo exposed to radiation (absorbed dose: 2 Gy); RAD + SFN: lung tissue from SFN-pretreated rats ex vivo exposed to radiation (absorbed dose: 2 Gy); RAD + CUR: lung tissue from CUR-pretreated rats ex vivo exposed to radiation (absorbed dose: 2 Gy). Statistical analysis: one-way ANOVA + Tukey’s post hoc multiple-comparison test. Statistical significance is denoted by asterisks: * *p* < 0.05. Data are presented as mean ± SD. Bars represent mean values and error bars indicate ± SD. *n* = 5 independent biological samples per group. Horizontal lines indicate the specific pairwise comparisons between the groups; comparisons without connecting lines are not statistically significant.

The calculated scores of oxidative stress and antioxidant protection are shown in Figure 2.

Irradiation (RAD) significantly increased the D score compared with the control group (*p* < 0.05) and was also significantly higher than in the CUR-treated group (*p* < 0.05). Within irradiated groups, RAD + CUR exhibited a significantly lower D score compared with RAD alone (*p* < 0.05).

For the OXY score, irradiation (RAD) significantly decreased values compared with the control group (*p* < 0.05). Among irradiated groups, RAD + CUR showed a significantly higher OXY score compared with RAD alone (*p* < 0.05).

For P score, there were no statistical differences in any of the treated groups.

**Figure 3:**
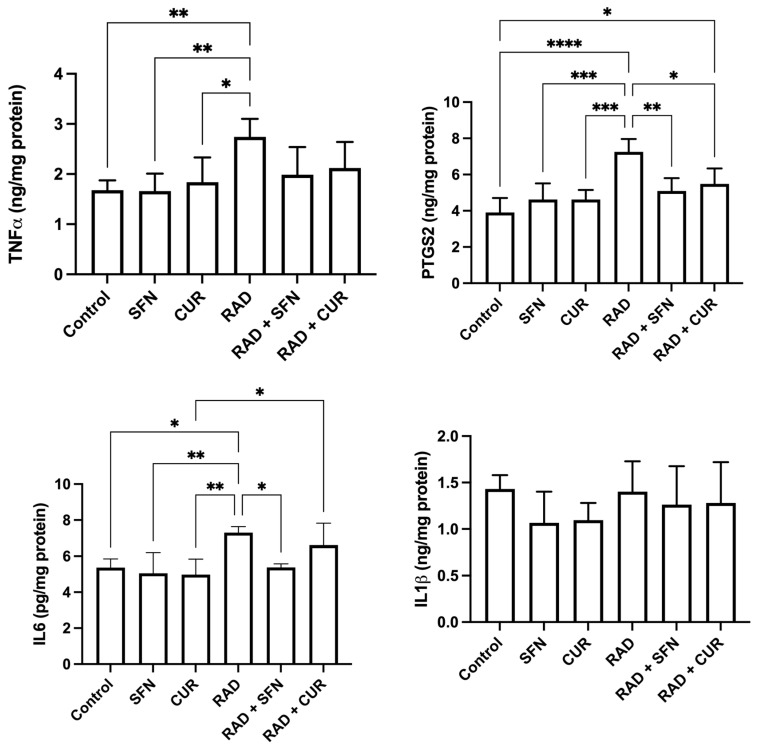
The effect of a single exposure of rat lung tissue to ionizing radiation on parameters of inflammation, as well as the protective effects of sulforaphane (SFN) and curcumin (CUR) administered per os for 28 days prior irradiation. Results are expressed per mg of protein (Bradford assay, bovine serum albumin standard). Control: lung tissue from vehicle-treated rats (5% DMSO, 28 days); SFN: lung tissue from rats pretreated with SFN (2 mg/kg/day, 28 days); CUR: lung tissue from rats pretreated with CUR (4.13 mg/kg/day, 28 days); RAD: lung tissue from control rats ex vivo exposed to radiation (absorbed dose: 2 Gy); RAD + SFN: lung tissue from SFN-pretreated rats ex vivo exposed to radiation (absorbed dose: 2 Gy); RAD + CUR: lung tissue from CUR-pretreated rats ex vivo exposed to radiation (absorbed dose: 2 Gy). Statistical analysis: one-way ANOVA + Tukey’s post hoc multiple-comparison test. Statistical significance is denoted by asterisks: * *p* < 0.05, ** *p* < 0.01, *** *p* < 0.001, and **** *p* < 0.0001. Data are presented as mean ± SD. Bars represent mean values, and error bars indicate ± SD. *n* = 5 independent biological samples per group. Horizontal lines indicate the specific pairwise comparisons between the groups; comparisons without connecting lines are not statistically significant.

The measured inflammatory markers are shown in Figure 3. TNF-α levels were significantly increased in the RAD group compared with the control (*p* < 0.01) and relative to SFN- or CUR-treated animals alone (*p* < 0.05–0.01). No significant differences were observed among irradiated groups.

PTGS2 levels were significantly increased in the RAD group compared with the control (*p* < 0.0001) and compared with SFN- or CUR-treated animals alone (*p* < 0.001). Within irradiated groups, both RAD + SFN and RAD + CUR showed significantly lower PTGS2 levels compared with RAD alone (*p* < 0.01 and *p* < 0.05, respectively), demonstrating mitigation by both antioxidants, with SFN stronger than CUR. A statistically significant increase in PTGS2 was detected in the RAD + CUR group compared to the control (*p* < 0.05). In contrast, PTGS2 levels in the RAD + SFN group did not differ significantly from control.

IL-6 levels were significantly increased in the RAD group compared with the control group (*p* < 0.05), SFN-treated animals (*p* < 0.01), and CUR-treated animals (*p* < 0.01). Among irradiated groups, RAD + SFN showed a significant decrease in IL-6 compared with RAD alone (*p* < 0.05), indicating mitigation by SFN. RAD + CUR group remained significantly increased compared with CUR alone (*p* < 0.05).

No statistically significant differences in IL-1β levels were observed among any of the treated groups.

## 4. Discussion

Natural compounds that protect tissues from radiation-induced oxidative stress, inflammation, and RILI are an emerging focus in radiobiology. CUR’s radioprotective effects, which are mainly centred around reducing oxidative stress, inflammation, and fibrosis, are supported by several animal studies [5,19,20,21], whereas SFN remains far less explored in this context. To address this gap, our study directly compared CUR and SFN pretreatment under controlled in vivo/ex vivo conditions to assess their protective effects against radiation-induced oxidative damage and inflammation in rat lung tissue.

We analysed key markers of oxidative stress, antioxidant response, and composite damage scores. Subsequently, selected inflammation markers were measured. The oxidative stress profile observed in our study reflects the expected impact of radiation on lung tissue. Excessive generation of reactive oxygen and nitrogen species during irradiation leads to oxidative stress. This primarily affects membrane lipids and initiates lipid peroxidation, a key mechanism in disease pathogenesis. Because reactive radicals are difficult to quantify directly in vivo, MDA, a stable end product of lipid peroxidation, is widely used as a reliable biomarker of oxidative membrane damage [33]. In the current study, MDA was significantly increased after radiation, suggesting oxidative injury. SOD is a widely distributed antioxidant enzyme that protects cells from oxidative damage by catalysing the conversion of superoxide anions into molecular oxygen and hydrogen peroxide [34]. In our study, SOD showed a non-significant trend toward elevated activity in the irradiated group, likely reflecting an adaptive response to increased ROS. This is comparable to a study conducted on murine model, where γ-irradiation at doses in the range of 1–8 Gy increased both parameters, with significant changes in SOD observed only at the highest dose (which is 4 times higher than the dose applied in our study), whereas MDA showed statistically significant elevations at doses above 1.5 Gy [35]. Furthermore, in our study, SH groups, non-enzymatic thiol-based antioxidants involved in maintaining redox balance, were reduced after irradiation, consistent with their depletion during oxidative stress. For all -SH-containing enzymes examined to date, their loss of activity in solution after irradiation can be explained primarily by damage to these thiol groups [36]. GSH is a widely distributed tripeptide that plays a central role in cellular homeostasis, primarily through its antioxidant function in maintaining redox balance. It directly reacts with electrophilic compounds through nucleophilic interactions, forming thioether GSH S-conjugates [37]. In contrast, IMA represents another end product of oxidative stress, formed when excessive free radical production alters the N-terminal region of serum albumin, making it a sensitive marker of ischemic injury [38]. In our study, GSH and IMA levels remained unchanged across all the treated groups compared with the control, which may reflect the relatively moderate radiation dose used.

CUR effectively attenuated all irradiation-induced alterations observed in the present study. In irradiated lung tissue, CUR pretreatment resulted in a marked reduction in MDA levels compared with radiation alone, indicating a stronger protective effect against lipid peroxidation. A similar pattern was observed for total SH groups, as CUR pretreatment significantly preserved thiol levels in irradiated tissue compared with the RAD group, maintaining values closer to those of the control group. In contrast, SFN pretreatment resulted in only the partial preservation of total SH group levels. Although SH levels in the RAD + SFN group remained significantly lower than those in the control group, the extent of reduction was less pronounced than in the irradiated-only group, indicating a limited protective effect against thiol depletion. Furthermore, while MDA levels in the RAD + SFN group were numerically closer to control values, no statistically significant differences were observed when compared with the RAD group. These findings suggest that, under the present experimental conditions, SFN exerted modest protective effects on selected oxidative stress parameters, whereas the effects of CUR were more pronounced.

Exposure of isolated rat lung tissue to ionizing radiation caused a significant increase in DS and a marked decrease in the OXY score compared to the control, reflecting enhanced oxidative damage. This can be associated with aforementioned significantly elevated MDA levels and decreased SH groups, while SOD showed a non-significant increasing trend, indicating that this enzyme was activated but insufficient to counteract the radiation-induced stress. Hence, the PS remained largely unchanged, suggesting that the antioxidant system did not adequately respond to the increased damage. Pretreatment with CUR or SFN prevented the radiation-induced rise in DS and the decline in OXY, restoring these scores to levels comparable with the control group, suggesting protection against changes in these oxidative stress markers. Notably, in the case of both DS and OXY score, the effect was significant for RAD + CUR compared with RAD alone, whereas RAD + SFN did not differ significantly from RAD, indicating a stronger protective effect of CUR on these oxidative stress markers.

Elevated ROS not only drive lipid, DNA, and protein oxidation but also activate pro-inflammatory pathways, highlighting the close interconnection between oxidative stress and inflammatory responses [39]. For instance, it has been demonstrated that wild-type mice develop double-strand breaks, oxidatively clustered DNA lesions, and apoptosis in tissues distant from the irradiation site, whereas these effects are markedly reduced in immunodeficient models, highlighting the key role of the immune system in systemic genotoxic responses after localized irradiation [40]. At the molecular level, radiation-induced mitochondrial ROS in macrophages activate MAPK and NF-κB signalling pathways, driving an increased expression of pro-inflammatory cytokines such as TNF-α and IL-6, thereby linking oxidative stress to innate immune activation [41]. In addition, excessive ROS generation by polymorphonuclear neutrophils at sites of inflammation can impair endothelial function, promote the opening of intercellular junctions, and enhance leukocyte transmigration, thereby contributing to further tissue damage [42]. Hence, considering this well-established mechanistic link between oxidative stress and inflammation, and the fact that radiation induces a multifaceted inflammatory response in the lungs mediated by cytokines and enzymatic pathways, we selected four biomarkers to capture the key oxidative and inflammatory events. These biomarkers were used to evaluate the protective effects of SFN and CUR. TNF-α is a proinflammatory cytokine that triggers a cascade involving IL-6 and IL-1, promotes fibroblast growth, and increases after radiation, contributing to radiation pneumonitis. IL-6, produced by lung cells, regulates inflammation and acute-phase protein release; higher IL-6 levels are seen in irradiated lungs, and IL-6 deficiency reduces acute alveolar damage [43]. IL-1β is the primary cytokine rapidly induced following irradiation, even at doses as low as 1 Gy [44]. Prostaglandin Endoperoxide Synthase 2 (PTGS2/COX-2) is one of the most important inflammatory mediators activated following exposure to ionizing radiation in some organs. This enzyme plays a key role in the release of prostaglandins and cytokines, linking inflammation to oxidative injury [45]. In the present study, the irradiation of isolated rat lung tissue induced a clear pro-inflammatory response, as evidenced by the elevation of TNF-α, PTGS2, and IL-6. Pretreatment with CUR and SFN attenuated this response to varying degrees, highlighting their protective effects. Specifically, PTGS2 levels were elevated following irradiation; however, this increase was not observed in the RAD + SFN group compared with the control. Pretreatment with CUR partially reduced PTGS2 levels, whereas SFN showed a stronger suppressive effect. Additionally, significantly lower PTGS2 levels were observed in the RAD + SFN and RAD + CUR groups compared with RAD alone, demonstrating a protective effect against this inflammatory marker. Notably, this reduction was more pronounced in the SFN-pretreated group, suggesting that SFN was more effective than CUR in modulating PTGS2 levels. IL-6 induction was attenuated by both CUR and SFN pretreatment, although only SFN significantly reduced IL-6 levels in irradiated lung tissue compared with the RAD group. This finding suggests that SFN more effectively mitigates this component of the radiation-induced inflammatory response in lung tissue. Although both CUR and SFN pretreatment prevented a statistically significant increase in TNF-α relative to the control, no significant differences were observed compared with the irradiated tissue alone, suggesting that TNF-α was less responsive to these treatments under the conditions tested. The lack of significant change in IL-1β might suggest that the absence of immune cell infiltration in ex vivo model limits inflammasome activation and secretion of mature IL-1β.

In published literature, CUR was examined for its protective effects on IL-4, IL-13, DUOX1, and DUOX2 expression and on lung histopathology after a higher radiation dose than in our study (15 Gy). Radiation increased IL-4, IL4Ra1, DUOX1, and DUOX2, promoting oxidative stress and fibrosis, whereas CUR, also applied at a higher dose than in our study (150 mg/kg) but over a shorter regimen (from 1 day before to 3 days after irradiation), reduced gene expression and lessened lung alterations [5]. This study supports our findings by showing that CUR reduces radiation-induced oxidative stress and inflammation. Furthermore, it was investigated whether CUR (200 mg/kg body weight/day prior to a single irradiation and for 8 weeks after radiation (a single 18 Gy dose)) could mitigate radiation-induced macrophage accumulation, interstitial oedema, alveolar septal thickness, perivascular fibrosis, and lung collapse. The results also demonstrated that prolonged CUR administration reduced the expression of TGF-β1, CTGF, TNF-α, TNFR1, and COX-2, while inhibiting NF-κB activation [19]. These results align with ours, as CUR also reduced TNF-α– and COX-2–related inflammatory responses, which we similarly found to be lowered after pretreatment. A study has also demonstrated the efficacy of CUR (1% or 5% CUR weight/weight) as a preventive agent in radiation-induced (13.5 Gy) pneumonopathy and in regression of lung tumours in a mouse model. In vitro, CUR enhanced antioxidant defenses by increasing HO-1 and reducing radiation-induced ROS. In vivo, it raised HO-1 levels within a week, reduced LPS-induced TNF-α at both doses, and—at 5%—improved survival and decreased lung fibrosis. It also did not protect lung tumour metastases from radiation, indicating lung protection without tumour radioprotection [20]. A study has demonstrated that liposomal CUR inhibited NF-κB activation, reduced irradiation-induced inflammatory cytokines, enhanced intratumoral apoptosis and microvessel responses, significantly suppressed tumour growth in LL/2 lung carcinoma, mitigated lung fibrosis, and showed no observable toxicity over a longer period than in our study (180 days) in mice irradiated with single dose of 14 Gy [21]. Only a single study, however, explored protective effect of SFN against radiation-induced lung damage. In that study, mice received 12 Gy thoracic irradiation with or without SFN (3–10 mg/kg). It was shown that SFN treatment markedly reduced radiation-induced lung injury by lowering IL-6, TNF-α, TGF-β1, suppressing NLRP3/NF-κB activation, and improving lung pathology in a dose-dependent manner [14]. Their results closely match ours, as SFN decreased IL-6 and TNF-α after irradiation, mirroring the complete IL-6 suppression and reduced TNF-α we observed. In a review article, Nrf2 was identified as a promising, yet still insufficiently explored, therapeutic target for the prevention and treatment of radiation-induced lung injury. Owing to its capacity to attenuate inflammation, oxidative stress, fibrosis, and cell death, Nrf2 activation was proposed as a potentially effective strategy, underscoring the need for more detailed mechanistic investigations and stronger clinical evidence [46]. This is important having in mind that SFN is a well-established activator of Nrf-2 [12].

Hence, although CUR’s radioprotective effects have been investigated, studies remain limited and mechanistic data are still scarce, while the role of SFN in radiation-induced lung injury is even less explored, highlighting the need for a direct comparative study of both compounds. Based on the parameters measured in our study, SFN showed greater protection against radiation-induced inflammation, while CUR was more effective in reducing oxidative stress, suggesting that these agents may act through complementary mechanisms to safeguard lung tissue from radiation-induced injury. Hence, future studies could investigate whether combined application of SFN and CUR produces additive or synergistic effects, as suggested by their distinct effects on different markers in the present study.

The current study has several limitations that should be considered when interpreting the results. It is important to acknowledge that oxidative and inflammatory alterations can also be driven by underlying pathological conditions [47], independent of radiation exposure, and are not solely attributable to radiation exposure. In the lung, oxidative stress generated by endogenous processes or exogenous stressors, such as cigarette smoke, environmental pollutants, or other toxic insults, can trigger inflammation and contribute to the development and progression of chronic airway diseases, including asthma, chronic obstructive pulmonary disease (COPD), and lung cancer [48]. Among these stressors, exposure to radiation represents an additional factor that can exacerbate oxidative damage and inflammatory responses in lung tissue. Furthermore, the use of isolated lung tissue excludes systemic interactions, immune cell recruitment, and vascular contributions, which are important in the pathogenesis of radiation-induced lung injury. Furthermore, a single radiation dose was applied, limiting the assessment of cumulative or fractionated exposure effects that more closely mimic clinical radiotherapy. In addition, due to the limited sample size, this study focused on acute oxidative stress and inflammatory markers, without evaluating long-term outcomes, such as lung tissue fibrosis. Finally, the relatively low dose of curcumin used in this study (4.13 mg/kg b.w./day) should be acknowledged. Curcumin has well-established antioxidant properties; however, its oral bioavailability is limited, as only approximately 60% of an administered dose is absorbed, and the compound is rapidly metabolized and excreted, resulting in low circulating levels of the parent molecule [49]. Oral delivery can be optimized by improving solubility using ambivalent solvents, such as DMSO, which allows consistent dosing and tissue exposure without fundamentally altering pharmacokinetics [50]. In the present study, curcumin was dissolved in DMSO to ensure reliable oral administration. Despite its limited systemic availability, multiple rodent studies have shown that orally administered curcumin exerts pronounced biological and radioprotective effects. For example, dietary curcumin at 5% (*w*/*w*) in mice achieved steady-state plasma concentrations in the range of 20–25 μg/mL (54–68 μM) by day 7, enhanced pulmonary antioxidant defenses including induction of heme oxygenase-1 (HO-1), reduced radiation-induced pneumonopathy, and improved survival, without protecting tumour cells [20]. Similarly, oral curcumin administered at 400 μmol/kg for five consecutive days around γ-irradiation significantly reduced cytogenetic damage, oxidative stress markers (TBARS, hydroperoxides, xanthine oxidase), and apoptotic signalling while restoring glutathione and antioxidant enzyme activities (SOD, CAT, GPx) in irradiated mice [51]. Evidence from metabolic disease models further supports the biological efficacy of moderate oral doses: in STZ-induced diabetic rats, curcumin administered at 15–30 mg/kg for 2 weeks improved renal function and attenuated oxidative stress by decreasing lipid peroxidation and restoring glutathione and antioxidant enzyme activities (SOD, CAT) compared with untreated diabetic rats [52]. In the present study, the selected dose corresponds to amounts commonly found in commercially available dietary supplements in Serbia, enhancing the translational relevance of our findings and enabling an evaluation of radioprotective effects at realistic, population-relevant exposure levels. The 28-day pretreatment with CUR and SFN was designed to establish a stable antioxidant and anti-inflammatory status in lung tissue prior to radiation exposure, thereby allowing the assessment of their protective potential. Prolonged administration enables sustained modulation of redox-sensitive pathways. This includes enhancing endogenous antioxidant defenses and reducing basal inflammatory responses. Both of these effects are critical determinants of tissue susceptibility to ionising radiation–induced injury. This duration allows sufficient time for biochemical and molecular adaptations to develop and minimizes transient effects associated with acute dosing at the time of irradiation.

## 5. Conclusions

Our study demonstrated that ionizing radiation induced oxidative and inflammatory changes in lung tissue, evidenced by increased MDA, elevated SOD activity, reduced SH groups, higher DS and lower OXY score, and upregulation of IL-6, and PTGS2. Both CUR and SFN provided protective effects against radiation-induced changes in measured oxidative stress and inflammatory markers in lung tissue. CUR effectively mitigated oxidative stress by attenuating MDA accumulation and preserving SH groups, while SFN showed stronger anti-inflammatory effects, reducing IL-6, and PTGS2 induction. Both agents prevented the radiation-induced rise in composite damage and oxidative stress scores, restoring these scores to levels comparable with the control group. These results indicate that CUR and SFN provide radioprotection through partially distinct but complementary mechanisms. CUR had a stronger effect on the measured oxidative stress–related parameters, while SFN more prominently affected inflammatory markers. Together, these findings suggest that their combined effects warrant investigation in future studies. To the best of our knowledge, this study is the first to directly compare the radioprotective effects of CUR and SFN in rat lung tissue, providing novel insight into their distinct yet complementary mechanisms in mitigating the observed radiation-induced oxidative stress and inflammatory changes, and laying the groundwork for future investigations of their potential combined use.

## Figures and Tables

**Table 1:** Methodology for the determination of oxidative stress/antioxidant defence parameters in rat lung tissue.

Parameter	Method Principle	Units	Reference
Malondialdehyde (MDA)	TBARS assay: absorbance of MDA and TBA-reactive substances after heating (100 °C, 5 min), cooling (10 min), centrifugation (10,000 *g*, 4 °C, 10 min)	µmol/mg protein	[27]
Ischemia-modified albumin (IMA)	Reduced cobalt-binding capacity of albumin after ischemia; reaction with cobalt and dithiothreitol; absorbance measured at 470 nm	ABSUs	[28]
Total sulfhydryl groups (SH)	Absorbance of yellow dianion (TNB2-) formed in alkaline medium with DTNB (pH 9.0)	mmol/mg protein	[29]
Glutathione (GSH)	Same principle as SH groups, after protein precipitation with 5% sulfosalicylic acid	µmol/mg protein	[29]
Superoxide dismutase (SOD)	Inhibition of adrenaline auto-oxidation at pH 10.2	% inhibition (U/mg protein)	[30]
Protein concentration	Coomassie Brilliant Blue G-250 dye-binding assay; absorbance shift from 465 to 595 nm; BSA standard	mg	[31]

## Data Availability

Data will be available on request.

## Abbreviations

The following abbreviations are used in this manuscript:

CUR	Curcumin
SFN	Sulforaphane
RILI	Radiation-induced lung injury
ROS	Reactive oxygen species
MDA	Malondialdehyde
SOD	Superoxide dismutase
SH	Sulfhydryl groups
GSH	Reduced glutathione
IMA	Ischemia-modified albumin
DS	Damage score
OXY	Oxidative stress score
PS	Protection score
TNF-α	Tumour necrosis factor alpha
IL-6	Interleukin 6
IL-1β	Interleukin 1 beta
PTGS2	Prostaglandin-endoperoxide synthase 2
COX-2	Cyclooxygenase 2
NF-κB	Nuclear factor kappa B
Nrf2	Nuclear factor erythroid 2-related factor 2
Keap1	Kelch-like ECH-associated protein 1
HO-1	Heme oxygenase 1
NLRP3	NOD-like receptor family pyrin domain containing 3
TGF-β1	Transforming growth factor beta 1
CTGF	Connective tissue growth factor
TNFR1	Tumour necrosis factor receptor 1
DUOX1	Dual oxidase 1
DUOX2	Dual oxidase 2
LPS	Lipopolysaccharide
